# ﻿A new combination in *Pseudolappula* (Boraginaceae, Rochelieae) based on morphological, molecular and palynological evidence

**DOI:** 10.3897/phytokeys.187.75346

**Published:** 2021-12-16

**Authors:** Dan-Hui Liu, Xue-Min Xu, Yi He, Quan-Ru Liu

**Affiliations:** 1 Key Laboratory of Biodiversity Science and Ecological Engineering, Ministry of Education, College of Life Sciences, Beijing Normal University, Beijing 100875, China. Beijing Normal University Beijing China

**Keywords:** Boraginaceae, *Lappulaoccultata*, new combination, *Pseudolappula*

## Abstract

*Lappulasinaica* was recently transferred to the monotypic genus *Pseudolappula* based on phylogenetic studies, while the related species, *L.occultata*, has remained in the genus *Lappula*. In this study, morphological, molecular, and palynological evidence supports that *L.occultata* should be transferred to the genus *Pseudolappula*. Both *L.occultata* and *P.sinaica* share a combination of nutlets features that distinguish them from *Lappula*: a longer adaxial keel and a linear attachment scar. Phylogenetic analysis based on ITS and *trnL-F* strongly supports *L.occultata* as the sister taxon of *P.sinaica*. In addition, pollen grains of these two species are 3-syncolporate with 3 alternating pseudocolpi, which is significantly different from the grains of *Lappula* taxa. Based on the above evidence, the new combination *Pseudolappulaoccultata* is proposed.

## ﻿Introduction

Recent phylogenetic studies on the Rochelieae (Boraginaceae) have greatly advanced our understanding of this plant group, and the circumscription of some genera has been changed (Huang et al. 2013; Saadati et al. 2017; Khoshsokhan-Mozaffar et al. 2018), the genus *Lappula* being one of these. Phylogenetic analyses indicate that *Lappula* is not monophyletic, with the species of this genus placed in three different lineages (Khoshsokhan-Mozaffar et al. 2018). The systematic position of *Lappulasinaica* (A.DC.) Asch. & Schweinf. was distinctive in occurring on a separate branch of the subtribe Eritrichiinae, while the other taxa of *Lappula* were clustered in different clades. After considering both molecular results and morphological comparisons, Khoshsokhan-Mozaffar et al. (2018) transferred the species *L.sinaica* to a new monotypic genus, *Pseudolappula*.

*Echinospermumsinaicum* A.DC. was described by Candolle (1846), based on two collections from the Sinai Peninsula, Egypt. This species was subsequently transferred to the genus *Lappula* by Ascherson and Schweinfurth (1887) and has since been regarded as a member of that genus (Candolle 1846; Gürke 1894; Brand 1931; Popov 1953; Riedl 1967; Wang 1989; Zhu et al. 1995; Ovczinnikova 2005a, 2009; Ovchinnikova et al. 2017).

The related species, *Lappulaoccultata*Popov (1951), was described based on specimens from Sary-tau mountains, Tajikistan. The type specimen of this species was designated by Ovchinnikova et al. (2020). In the protologue, the author stated that *L.occultata* differs from *L.sinaica* in its erect pedicel and long calyx. Both *L.sinaica* and *L.occultata* share morphological features of the nutlets and the two species have been viewed as sister taxa by most authors (Popov 1953; Riedl 1967; Wang 1989; Ovczinnikova 2005a, 2009). From 1953 to 2009, these two species were classified under the same section, subsection, and series. The systematic position of *L.sinaica*, *L.occultata*, and their congeneric relatives is presented in Table 1.

**Table 1.** Historical classifications of *L.sinaica* with *L.occultata* and its congeneric relatives.

	Popov (1953)	Riedl (1967)	Wang (1981)	Ovczinnikova (2005a)	Khoshsokhan-Mozaffar et al. (2018)
Genus	*Lappula*	*Lappula*	*Lappula*	*Lappula*	*Pseudolappula*
Section	*Eulappula*	*Lappula*	*Lappula*	*Sinaicae*	–
Series/Subsection	*Sinaicae*	*Sinaicae*	*Sinaicae*	–	–
Species	*L.sinaica*	*L.sinaica*	*L.sinaica*	*L.sinaica*	*P.sinaica*
–	*L.occultata*	*L.sessiliflora*	*L.occultata*	*L.occultata*	–
–	*L.lipschitzii*	–	–	*L.mogoltavica*	–

Although *L.sinaica* has now been formally placed in the new genus *Pseudolappula* by Khoshsokhan-Mozaffar et al. (2018), the related species, *L.occultata*, has remained in the genus *Lappula*. Current phylogenetic studies do not support the two taxa as allied species, as *L.sinaica* forms a distinct monospecific clade, while *L.occultata* is nested in the *Lappula* clade (Huang and Zhang 2012; Huang et al. 2013; Khoshsokhan-Mozaffar et al. 2018). Because of this obvious conflict between previous taxonomic treatments for *L.occultata* and the aforementioned molecular studies, further examination of these two species is needed, including past voucher specimens used.

The specimens used in the previous phylogenetic analyses (Huang et al. 2013; Khoshsokhan-Mozaffar et al. 2018) were examined. After comparing these with both the protologue and type specimens, we discovered that almost all the specimens of *L.occultata* preserved in Chinese herbaria were misidentified. In the protologue (Popov 1951), *L.occultata* is described has bearing small flowers, with the corolla limb 1–1.5 mm wide. However, in *Flora Reipublicae Popularis Sinicae* (Wang 1989) and *Flora of China* (Zhu et al. 1995), this species is described as having large flowers, with the corolla limb 5–6 mm wide, indicating a different, possibly incorrect, circumscription of *L.occultata* in Chinese Flora, and this may cause misunderstanding of *L.occultata*.

Therefore, it was necessary to revise the circumscription of *L.occultata* based on a more appropriate understanding and identification of that species. In this study, morphological, molecular, and palynological analyses are conducted to clarify the systematic position of *L.occultata*.

## ﻿Materials and methods

### ﻿Morphological observation

Specimens at PE, XJU, XJA, XJBI, MW, NSK, and TASH were examined critically, including type specimens. Field observations were carried out in Xinjiang province, China. Morphological studies were made of living plants in the field and of pressed specimens, with particular attention to the mature nutlets which were photographed by a ZEISS V8 stereoscopic microscope.

### ﻿Molecular taxon sampling

In order to verify the evolutionary relationships between *Pseudolappula* and *L.occultata*, 46 taxa within the tribe Rochelieae were sampled, including 5 genera of subtribe Eritrichiinae (*Hackelia*, *Pseudolappula*, *Eritrichium*, *Rochelia*, and *Lappula*), which covered all main clades of this lineage. *Pseudoheterocaryumsubsessile* (Vatke) Kaz.Osaloo & Saadati was selected as an outgroup according to the previous studies (Huang et al. 2013; Chacón et al. 2016; Khoshsokhan-Mozaffar et al. 2018). The DNA sequences obtained from this study were deposited in GenBank, with all accession numbers listed in Appendix 1.

### ﻿DNA extraction, amplification, and sequencing

Genomic DNA was extracted from silica-gel dried leaves using the Plant Genomic DNA Kit (Tiangen, Beijing, China), following the manufacturer’s instructions. The ITS (White et al. 1990; Sang et al. 1995) and *trnL-F* (Taberlet et al. 1991) regions were amplified using primer pairs of the cited authors. The amplification profile followed Huang et al. (2013). Products of amplification reactions were sequenced with an ABI3730XL automated DNA sequencer (BGI Tech. Solutions Beijing Liuhe Co., Limited, Beijing, China).

### ﻿Sequence alignment Phylogenetic analysis

Sequences of ITS and *trnL-F* were aligned with MAFFT online version 7 and manually adjusted (Katoh et al. 2019). A combined matrix of ITS and *trnL-F* was generated by SequenceMatrix (Vaidya et al. 2011). Combinability of ITS and *trnL-F* were assessed using the incongruence length difference test (Farris et al. 1994), as implemented in PAUP*4.0 (Swofford 2002). According to jModeltest (Darriba et al. 2012), the best evolutionary model was TrN+I+G. Molecular phylogeny reconstruction was performed in the CIPRES Science Gateway (Miller et al. 2015) with Maximum Likelihood analysis employing RAxML-HPC2 on XSEDE (8.2.12) (Stamatakis 2014) and Bayesian inference using MrBayes on XSEDE (3.2.7a) (Ronquist et al. 2012). For MrBayes, four Markov chains were run for 50,000,000 generations. The trees were sampled every 1,000 generations, while the first 25% of trees were discarded as burn-in. The rest of the trees were used to generate a majority-rule consensus tree. Trees were visualized using Fig. Tree v. 1.4.4.

### ﻿Pollen sampling and scanning electron microscopy

Samples of taxa were obtained from field surveys during 2019–2021 and from voucher specimens were preserved in the BNU herbarium (Appendix 2). Pollen grains of these samples were mounted on metallic stubs with conductive adhesive tape, then coated with gold using an E-1045 ion sputter. The prepared samples were observed with a Hitachi S-4800 scanning electron microscope at 5 kV. Terminology follows Punt et al. (2007) and Ovczinnikova (2021).

## ﻿Results

### ﻿Morphological studies

**Figure 1.**
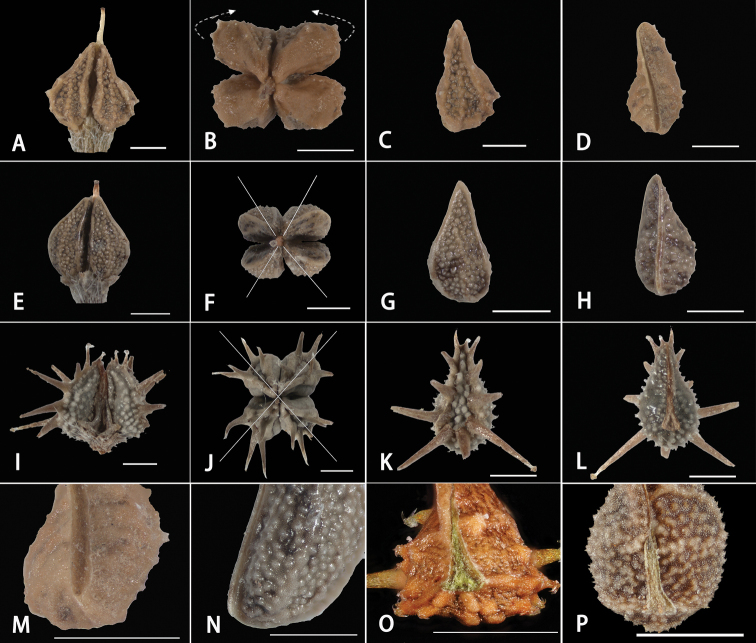
Nutlet morphology of *Pseudolappula* and *Lappula***A–D***Pseudolappulasinaica***A** fruit lateral view **B** fruit polar view (growth direction of nutlets indicated by arrows) **C** nutlet abaxial view **D** nutlet adaxial view **E–H***Lappulaoccultata***E** fruit lateral view **F** fruit polar view (arrangement of nutlets indicated by cross lines) **G** nutlet abaxial view **H** nutlet adaxial view **I–L***Lappulapatula***I** fruit lateral view **J** fruit polar view (arrangement of nutlets indicated by cross lines) **K** nutlet abaxial view **L** nutlet adaxial view **M** adaxial keel of *P.sinaica* (adaxial view) **N** adaxial keel of *L.occultata* (lateral view) **O** attachment scar of *Lappulabalchaschensis***P** attachment scar of *Lappulabrachycentra*. Scale bars: 1 mm.

The results of morphological comparisons indicated that *Pseudolappulasinaica* (A.DC.) Khoshsokhan, Sherafati & Kaz.Osaloo and *L.occultata* are quite similar and likely closely related. The two taxa exhibited a special combination of characters in both the nutlet attachment scar and adaxial keel (Fig. 1), and these characters are congruent with the description of section Sinaicae (Ovczinnikova 2005a). Both *P.sinaica* and *L.occultata* possessed no obvious attachment scar on the nutlets (Figs 1D, H, M, N), and the shape of cicatrix was linear (Fig. 1N). In addition, the length of adaxial keel was the same as the nutlet (Figs 1D, H). However, taxa in *Lappula* developed an ovoid, triangular-ovoid or narrow lanceolate attachment scar (Figs 1L, O, P), and the adaxial keel was shorter than the nutlet (Figs 1L, O, P). Detailed comparison of *Pseudolappula*, *L.occultata* and *Lappula* is provided in Table 2.

**Table 2.** Comparisons of *Pseudolappula*, *L.occultata* and *Lappula*.

**Characters**		***Pseudolappula***	***L.occultata***	***Lappula***
Life form		annual	annual	annual, biennial, perennial
Leaves	basal leaves	petiolate	petiolate	sessile
Bracts	lower shape	lanceolata	narrowly ovate	leaflike or linear
Calyx	shape	oblong-linear	linear	linear, oblong
Corolla	color	blue	blue	blue, white
	size (mm)	2–3 × 1–2 (3)	2–2.5 × 1–1.5	2.5–4 × 1.5–12
	limb/tube (ratio)	ca. 1	ca. 1	ca. 1–3
Stamens	pollen aperture type	3-syncolporate 3 pseudocolpi	3-syncolporate 3 pseudocolpi	3-colporate 3 pseudocolpi
	polar axis	12.3–15 μm	13.6–15.5 μm	8.9–19.4 μm
	equatorial axis	4.3–5.6 μm	4.7–7.1 μm	2.2–9.1 μm
Style	–	exceeding the nutlets	exceeding the nutlets	exceeding or not the nutlets
Nutlets	disk shape	narrowly oblong	oblong	lanceolata to ovate
	attachment scar (cicatrix)	not obvious, linear	not obvious, linear	obvious, narrow lanceolata to ovate
	adaxial keel	as long as the nutlets	as long as the nutlets	shorter than the nutlets

### ﻿Phylogenetic analyses

**Figure 2.**
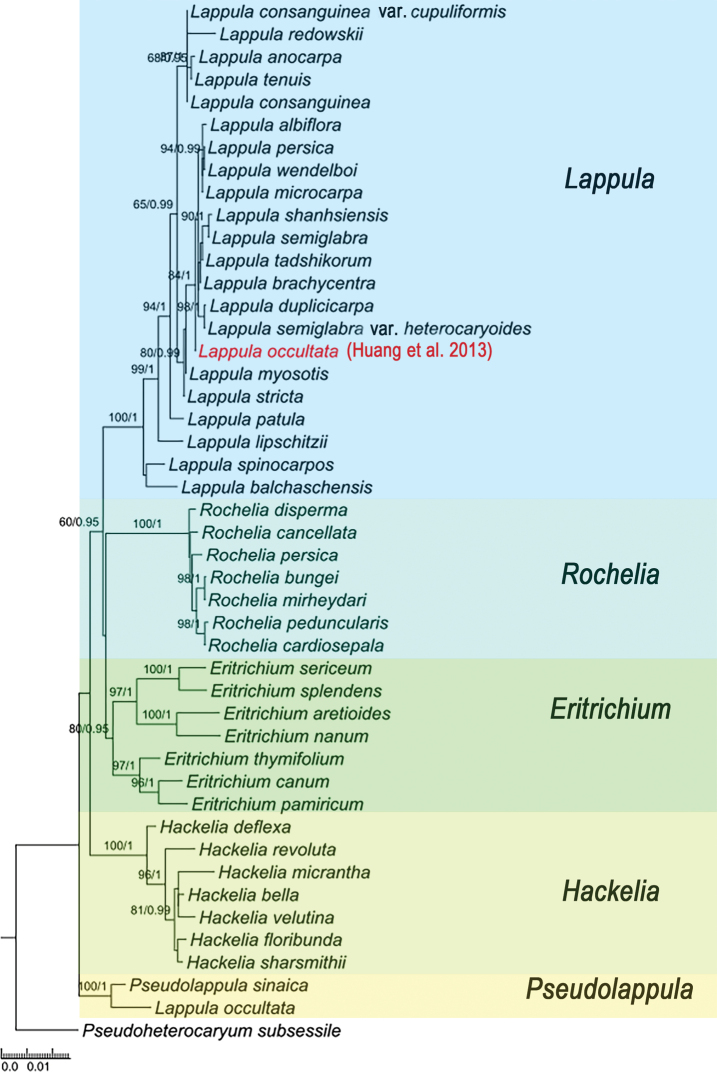
Maximum Likelihood tree of subtribe Eritrichiinae inferred from ITS + *trnL-F*. The tree topology was constructed using RAxML. Bootstrap values and Bayesian posterior probabilities are indicated above branches. Note that the *Lappulaoccultata* (Huang et al. 2013) is a misidentified voucher from previous studies (Huang et al. 2013).

The phylogeny was rooted with *Pseudoheterocaryumsubsessile* from the tribe Rochelieae, and the tree showed subtribe Eritrichiinae as monophyletic. Eritrichiinae comprised 5 major clades (Fig. 2). *Pseudolappula* was the first diverging clade (pp=1, ML-BS=100), followed by *Hackelia* (pp=1, ML-BS=100). *Lappula* resolved as sister to the clade that includes *Rochelia* and *Eritrichium*. Notably, the new sample of *L.occultata* from the present study was clearly clustered into a monophyletic clade with *P.sinaica* (Fig. 2), which was consistent with our morphological analysis.

### ﻿Palynological studies

**Figure 3.**
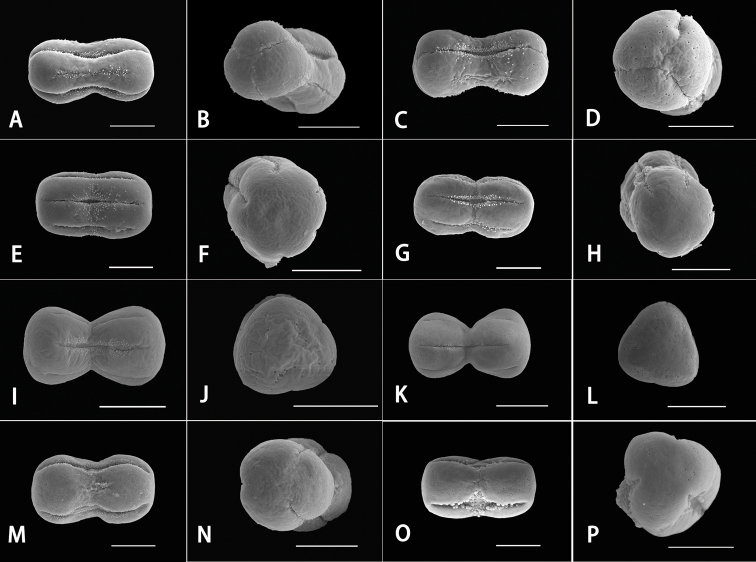
Scanning electron micrographs. **A, B***Pseudolappulasinaica***C, D***Lappulaoccultata***E, F***L.shanhsiensis***G, H***L.redowskii***I, J***L.macrantha***K, L***L.tianschanica***M, N***Rocheliabunge***O, P***Pseudoheterocaryumrigidum*. Scale bars: 5 μm.

The palynological data also supported that *P.sinaica* and *L.occultata* are closely related to each other. We examined the pollen morphology of *Pseudoheterocaryum*, *Pseudolappula*, *Lappula*, and *Rochelia* taxa. Pollen grains were isopolar, dumbbell-shaped or oblong in equatorial view and sub-circular in polar view (Fig. 3). Their sizes ranged from 8.9–19.4 × 2.2–9.1 μm.

The pollen apertures of the studied taxa were of three types: 3-colporate alternating with 3 pseudocolpi (Figs 3E, G, I, K, M), 3-syncolporate alternating with 3 pseudocolpi (Figs 3B, D) and 3-colporate types (Fig. 3O). Specifically, the true apertures of *P.sinaica* and *L.occultata* were 3-syncolporate, which is unique in the subtribe Eritrichiinae (Carr 1973; Díez and Valdés 1991; Mazari et al. 2018). The shapes of true apertures and pseudocolpi were narrowly linear. The length of pseudocolpi were nearly equal (Figs 3E, G, I, K) or shorter (Figs 3A, C, M) compared to colpi.

## ﻿Discussion

Nutlets are always important for identification and classification of Boraginaceae, especially for *Lappula* (Popov 1953; Riedl 1967; Zhu et al. 1995; Ovczinnikova 2005a, 2005b, 2006, 2009; Ovchinnikova et al. 2017, 2020). Traditionally, the classification of *Lappula* is heavily based on the abaxial characters of nutlets (number of rows of glochids, the length of glochids, the shape of the eremocarp disk, and the confluent degree of glochids). However, information on nutlets beyond well studied abaxial characters deserves more attention. *Pseudolappulasinaica* is such a case, although Khoshsokhan-Mozaffar et al. (2018) provided the key to distinguish *Pseudolappula* and *Lappula*. After examining the types and specimens for *P.sinaica* and species of *Lappula*, we consider these characters are not effective for recognizing each of the two genera. First, the length of nutlets between the two genera is overlapping (2 mm vs 2–5 mm). Second, the nutlets of *P.sinaica* possess a distinct margin (Fig. 1C) different from the description by Khoshsokhan-Mozaffar et al. (2018), whose use of immature nutlets may have led to differences and inaccurate descriptions. In addition, the character of the fruit pedicel is not reliable to differentiate the two genera, because not all the sampled individuals of *P.sinaica* have recurved pedicels on the herbarium specimens. Seemingly, some *Lappula* species, such as *L.semiglabra*, also have recurved pedicels. Even with the combined nutlets and pedicel features, it is challenging to distinguish the two genera.

*Lappulasinaica* and *L.occultata* were placed in the section Eulappula, series *Sinaicae* by Popov (1953) based on the nutlets characters, notably the adaxial keel. Since then, the systematic position of *L.sinaica* and its congeneric relatives has been relatively independent (Riedl 1967; Wang 1989; Ovczinnikova 2005a), and our results basically agree with previous treatments. Based on critical morphological comparisons, we find that the attachment scar and adaxial keel of nutlets are useful to separate *P.sinaica* and *L.occultata* from species in *Lappula*. Our morphological results are consistent with the findings of Ovczinnikova (2005a), who described the new section Sinaicae according to the above-mentioned characters. Additionally, the arrangement of 4 nutlets is unusual in both *L.occultata* and *P.sinaica* (Figs 1B, F). This arrangement pattern is in accordance with the view of Hilger (2014) concerning *Lappulaspinocarpos* (Forssk.) Asch. ex Kuntze, and some other researchers hold the view that *L.spinocarpos* should be separated from *Lappula* and raised to genus level (Brand 1931; Sadat 1989). Therefore, adaxial features of nutlets may be more critical than abaxial characters for the identification of *Pseudolappula*.

To better resolve relationships of *P.sinaica* with *L.occultata* and the *Lappula* species within subtribe Eritrichiinae, we newly sequenced the ITS and *trnL-F* regions from a ‘real’ specimen of *L.occultata*, which was determined to match the initial species description after careful specimen examination. As a result, the phylogenetic framework within tribe Rochelieae is highly congruent with previous work (Khoshsokhan-Mozaffar et al. 2018). However, our study cannot corroborate the recent authors’ treatment of *L.occultata* (Huang et al. 2013; Khoshsokhan-Mozaffar et al. 2018). Our phylogenetic result shows that *L.occultata* is more closely related to *P.sinaica* than to any member of the *Lappula*. The incongruous systematic position of *L.occultata* (Fig. 2) is based on misidentification due to the incorrect description of *L.occultata* in *Flora Reipublicae Popularis Sinicae* and *Flora of China*. Consequently, specimens of *Lappulabrachycentra* (Ledeb.) Gürke are incorrectly identified as *L.occultata*. Furthermore, we carefully examined *Lappula* specimens at the same location that Huang et al. (2013) sampled. These specimens possess very short marginal glochids and large flowers. In some individuals, there are no visible glochids, but only marginal ribs on the nutlets. These characters are more in line with the inaccurate descriptions of *L.occultata* in Chinese flora (Wang 1989; Zhu et al. 1995) and frequently cause misidentifications.

Boraginaceae are a palynologically heterogeneous family (Erdtman 1969; Nowicke and Miller 1990), and pollen grains also could be useful for lower taxonomic levels, such as genera (Bigazzi et al. 2006; Liu et al. 2010; Sutorý 2013; Noroozi et al. 2021). The subtribe Eritrichiinae is one of the major clades of tribe Rochelieae with 6 genera and over 200 species (Chacón et al. 2016), but palynological studies of this subtribe are insufficient. In the present research, pollen morphology of 8 taxa of *Pseudoheterocaryum*, *Pseudolappula*, *Lappula*, and *Rochelia* were studied. Their pollen grains are mostly heterocolpate, which is consistent with previous studies (Díez and Valdés 1991; Khatamsaz 2001). Specifically, the pollen apertures of *L.occultata* and *P.sinaica* are 3-syncolporate alternating with 3 pseudocolpi (Figs 3B, D), which is unique, and the characters of pollen apertures shared by these two species are not found in other members of subtribe Eritrichiinae (Carr 1973; Díez and Valdés 1991; Khatamsaz 2001; Mazari et al. 2018).

Although *Lappula* species have been studied in terms of nutlet morphology (Wu et al. 2014; Ovczinnikova 2006, 2021), palynology (Ahn and Lee 1986; Díez and Valdés 1991; Khatamsaz 2001), cytology (Löve 1975, 1983; Luque 1992; Kobrlová and Hroneš 2019), and phylogeny (Huang et al. 2013; Khoshsokhan-Mozaffar et al. 2013, 2018), there are still limits due to insufficient sampling. On the one hand, taxonomic and phylogenetic studies require very broad sampling. On the other hand, correct identification of species is fundamental for the various research. *Lappula* is a taxonomically difficult genus, and nutlet characters are essential for the proper identification of species in this genus. However, we must be careful not to rely too much on one feature for taxonomic delimitation. More characters should be closely investigated and integrated into further work.

## ﻿Taxonomic treatment

### ﻿ Pseudolappula occultata

Taxon classificationPlantaeBoraginalesBoraginaceae

(Popov) Q.R.Liu & D.H.Liu comb. nov.

D78B1E80-E93D-5C94-AE98-E3450545D469

urn:lsid:ipni.org:names:77234443-1

#### Basionym.

*Lappulaoccultata*Popov (1951: 331).

#### Type.

Tajikistan: Sary-tau mountains. 25 June 1920, *Popov 697* (TASH003719!).

#### Description.

Annual herbs. Stems erect, 15–40 cm tall, frequently branched from base or above middle, with appressed or semi-appressed white hairs. Basal leaves oblong with obvious petiole, 2–3 cm long, 5–8 mm wide; cauline leaves sessile, oblong to lanceolate, 2–4 cm long, 4–9 mm wide, with spreading hairs, hairs discoid at base. Inflorescences to 10–15 cm in fruit; bracts small, narrowly ovate to linear. Pedicels erect, the lower 5–6 mm long. Calyx lobes linear, erect, 2 mm long, to 4–5 mm in fruit, enclosing the nutlets. Corolla blue, 2–3 mm long, the tube shorter than calyx; throat appendages yellow, trapeziform, ca. 0.3 mm; limb 1–1.5 mm wide, lobes ovata-rounded. Stamen 5, filaments short, pollen grains isopolar, dumbbell-shaped in equatorial view and subcircular in polar view, 3-syncolporate apertures alternating with 3 pseudocolpi, with 6 orae. Coenobium 2–3 mm in diameter, homomorphic nutlets. Style surpassing the nutlets by ca. 0.5 mm. Nutlets ovoid, shiny, ca. 2 mm long, not easily separated from gynobase; disk ovate, weakly keeled, densely with rounded granulose, margin prominent and forming a narrow smooth rim. Cicatrix linear, not obvious, adaxial keel ca. 2 mm long.

#### Phenology.

Flowering and fruiting from May to July.

#### Distribution and habitat.

China, Kazakhstan, Tajikistan, Uzbekistan, Kyrgyzstan, Afghan, Mongolia (Vvedensky 1961; Sharashova 1962; Goloskokov 1964; Chukavina 1984; Ovczinnikova 2005a; Ovchinnikova et al. 2017). It grows on rocky slopes at elevations of 600–2400 m (Chukavina 1984).

#### Note.

The section Sinaicae (Riedl) Ovczinnikova was proposed by Ovczinnikova (2005a). This small section is comprised of 3 species, *L.sinaica*, *L.occultata*, and *Lappulamogoltavica* Popov ex Czukav. *L.sinaica* has been transferred to the new monotypic genus, and our study supports that *L.occultata* should also be combined into the genus *Pseudolappula*. Then, the systematic position of *L.mogoltavica* needs to be settled. The species *L.mogoltavica* was published by Czukavina (1983). Ovchinnikova et al. (2017) conducted a detailed study on this species and its congeneric relatives, and morphological comparisons demonstrate that the length of the adaxial keel and the shape of cicatrix are uniform in section Sinaicae. These characters suggest that the three species have a close relationship.

#### Additional specimens examined.

**China**. Xinjiang: Yining county, 29 April 1977, *suzenan 613* (XJU!); Gongliu county to Tekes county, 9 August 1996, *wangqi 96–047* (XJA00057876!); Nilak county, 43°41'18.28"N, 82°20'4.70"E, alt. 1287 m, 17 May 2020, *Dan-Hui Liu BNU2020XJ088* (BNU!); Huocheng county, 44°18'35.61"N, 81°6'6.07"E, 21 May 2021, *Dan-Hui Liu BNU2021XJ095* (BNU!). **Kazakhstan**. Alma-Ata region, Enbekshikazakh district, 43°39'N, 78°56'E, 17 May 2003, *A.Yu.Korolyuk*, *I.A.Khrustaleva s.n.* (NSK0005820!); East Kazakhstan region, Urdzhar district, alt. 660 m, 30 May 2003, *A.Yu.Korolyuk 45* (NSK0005868!); Karaganda region, 47°42994'N, 74°81770'E, 13 May 2014, *A.L.Ebel s.n.* (NSK0008730!); Baidibek district, 25 April 2015, *N.N.Lashchinsky s.n.* (NSK0008234!). **Uzbekistan.** Viloyat Surxondaryo, 15 km W from city Boysun, 38°14'12"N, 67°1'54"E, alt. 1140 m, 7 May 2013, *D.Lyskov s.n*. (MW0895220!). **Afghan.** Kabul, 20 km SE, alt. 2000–2400 m, 21 May 1968, *Freitag 2641* (KUFS!); Kapisa, Unteres Panjsher Tal gegenueber Korawa, alt. 1700 m, 24 May 1973, *O. Anders 9764* (KUFS!); Takhar, Gebirgshange 12 km SE Eshkamesh, alt. 1300–2100 m, 24 May 1970, *O. Anders 6751* (KUFS!); Upper Maidan-valley near Takona, alt. 2700 m, 11 June 1968, *Freitag 2970* (KUFS!); Loghar, Dobandaytal, alt. 2800 m, 4 May 1970, *O. Anders 3356* (KUFS!); Paktya, Saydkhelo Lgad, alt. 1620 m, 12 May 1972, *O. Anders 8877* (KUFS!); Nangahar, N Haenge des Safeed Koh bei Baghdara, alt. 1200–1400 m, 23 March 1973, *O. Anders 9511* (KUFS!).

## Supplementary Material

XML Treatment for Pseudolappula occultata

## ﻿Appendix 1

Taxa used for molecular analyses (Taxon, GenBank accession no. (ITS, *trnL-F*), source and collector/collection number).

**Outgroup Taxa**: ***Pseudoheterocaryumsubsessile*** Vatke, AB758297, AB758326, Iran, *Faghihna & Zangooei 28193* (TMUH). **Ingroup Taxa**: ***Eritrichiumaretioides*** DC. KU927709, KC542591, U.S.A., *Weigend 9126* (BSB); ***Eritrichiumcanum*** (Benth.) Kitam. AB758294, AB758323, Germany, cultivated in Munich Botanical Garden; ***Eritrichiumnanum*** Schrad. Ex Gaudin, KU927711, KC542483, Switzerland, *Zippel & al*. *s.n.* (B); ***Eritrichiumpamiricum*** B.Fedtsch. KU927712, KC542564, Afghanistan, *Anders 8098* (M); ***Eritrichiumsericeum*** DC. JQ388500, Russia, West Chukotka, *Petrovsky & Plieva s.n.* (O); ***Eritrichiumsplendens*** Kearney, JQ388501, Alaska, Noatak Quad, *Solstad & Elven 03/1216* (O); ***Eritrichiumthymifolium*** (DC.) Y.S.Lian & J.Q.Wang, JX976807, JX976913, China, Xinjiang, *B. C. Han et J. F. Huang 201007004* (PE); ***Hackeliabella*** I.M.Johnst., KU927715, KC542497, U.S.A., *Merello & al*. *702* (MO); ***Hackeliafloribunda*** I.M.Johnst., JQ513445, *Reveal 2390* (SD 103849) KC542513, USA *Miller et al. 6966* (MO) ; ***Hackeliamicrantha*** (Eastw.) J.L.Gentry, JQ388504, JQ388584, U.S.A, Oregon, Grant Co., *Hinchliff 869* (WS); ***Hackeliarevoluta*** I.M.Johnst., KF849119, KF849224, Argentina, *C. Aedo 15407* (MA); ***Hackeliasharsmithii*** I.M.Johnst., KU927717, KC542498, U.S.A., *Hilger U.S.A. 94/18* (BSB); ***Hackeliavelutina*** I.M.Johnst., KU927719, KC542530, U.S.A., *Hilger 411/1997* (BSB); ***Hackeliadeflexa*** (Wahlenb.) Opiz, JX976808, JX976914, China, Xinjiang, *J. F. Huang 20090109* (XJBI); ***Lappulaalbiflora*** (Riedl) Khoshsokhan & Kaz.Osaloo, KF287982, KF288065, *Rechinger 31424* (TARI); ***Lappulaanocarpa*** Ching J.Wang, JQ388505, JQ388585, China, Xinjiang, *Juan Qiu 08-0007* (XJA); ***Lappulabalchaschensis*** M.Popov ex Golosk., JX976776, JX976890, China, Xinjiang, *J. F Huang et B. C. Han 201008016* (XJBI); ***Lappulabrachycentra*** (Ledeb.) Gürke, JX976777, JX976891, China, Xinjiang, *J. F. Huang et B. C. Han 201008042* (XJBI); ***Lappulaconsanguinea*** (Fischer et C.A. Mey.) Gürke, JX976779, JX976893, China, Xinjiang, *J. F. Huang 20090213* (XJBI); ***Lappulaconsanguinea*** var. ***cupuliformis*** Ching J.Wang, JX976780, JX976894, China, Xinjiang, *J. F. Huang et B. C. Han 201009011-2* (XJBI); ***Lappuladuplicicarpa*** Pavlov, JX976781, JX976895, China, Xinjiang, *J. F. Huang 20090181* (XJBI); ***Lappulalipschitzii*** Popov, JX976787, JX976899, China, Xinjiang, *W. Zhai 087* (SHI); ***Lappulamicrocarpa*** (Ledeb.) Gürke, JX976788, JX976900, China, Xinjiang, *J. F. Huang et B. C. Han 201009003* (XJBI); ***Lappulamyosotis*** Moench, JX976789, JX976901, China, Shanxi, *J. F. Huang 2010050* (XJBI); ***Lappulaoccultata*** Popov, JX976791, JX976902, China, Xinjiang, *J. F. Huang et B. C. Han 201008043* (XJBI); OK135686, OL364176, China, Xinjiang, *Dan-Hui Liu BNU2020XJ088* (BNU); ***Lappulapatula*** (Lehm.) Menuharth, JX976792, JX976903, China, Xinjiang, *J. F. Huang 20090056-A* (XJBI); ***Lappulapersica*** (Boiss.) Khoshsokhan & Kaz. Osaloo, AB758312, AB758339, Iran, *Assadi and Maassumi 51278* (TARI); ***Lappularedowskii*** (Hornem.) Greene, KP027121; KF288063, U.S.A., *Cohen 161*; ***Lappulasemiglabra*** (Ledeb.) Gürke, JX976793, JX976904, China, Gansu, *J. F. Huang et B. C. Han 2010055* (XJBI); ***Lappulasemiglabra*** var. ***heterocaryoides*** Popov ex Ching J.Wang, JX976784, JX976896, China, Xinjiang, *J. F. Huang et B. C. Han 201008054* (XJBI); ***Lappulashanhsiensis*** Kitag., KU927725, KU927725, China, *Kürschner & al*. *634* (BSB); ***Lappulaspinocarpos*** (Forssk.) Asch. ex Kuntze, JX976795, JX976906, China, Xinjiang, *W. Zhai 120* (SHI); ***Lappulastricta*** (Ledeb.) Gürke, JX976798, JX976907, China, Xinjiang, *J. F. Huang et B. C. Han 201008052* (XJBI); ***Lappulatadshikorum*** Popov, JX976799, JX976908, China, Xinjiang, *W. Zhai 12* (SHI); ***Lappulatenuis*** (Ledeb.) Gürke, JX976800, JX976909, China, Xinjiang, J. *F. Huang et B. C. Han 201008049* (XJBI); ***Lappulawendelboi*** (Riedl) Khoshsokhan & Kaz.Osaloo, AB758314, AB758340, Iran, *Kazempour Osaloo 2008-7* (TMUH); ***Rocheliabungei*** Trautv., AB564695, AB564705, Iran, *Assadi & Massoumi 55785* (TARI); ***Rocheliacancellata*** Boiss. & Balansa, AB564702, AB564712, Turkey, *Bani 4971* (TMUH); ***Rocheliacardiosepala*** Bunge, AB564701, AB564711, Iran, *Kazempour Osaloo 2006-1* (TMUH); ***Rocheliadisperma*** (L.) Wettst., AB564698, AB564708, Iran, *Kazempour Osaloo 2007-2* (TMUH); ***Rocheliamirheydari*** Riedl & Esfand., AB564696, AB564706, Iran, Faghihnia & Zangooei 23477 (TMUH); ***Rocheliapeduncularis*** Boiss., AB564699, AB564709, Iran, Abdolzadeh 20447 (FUMH); ***Rocheliapersica*** Bunge ex Boiss., AB564697, AB564707, Iran, *Kazempour Osaloo 2007-1* (TMUH); ***Pseudolappulasinaica*** (A.DC.) Khoshsokhan, Sherafati & Kaz.Osaloo, AB758308, AB758336, Iran, *Kazempour Osaloo 2007-7* (TMUH);

## ﻿Appendix 2

**Table:** Sources of the pollen materials.

**Species**	**Voucher**
*Pseudoheterocaryumrigidum* (A.DC.) Kaz.Osaloo & Saadati	China. Xnjiang, *Dan-Hui Liu BNU2020XJ079* (BNU)
*Lappulamacrantha* (Ledeb.) Gürke	China. Xnjiang, *Dan-Hui Liu BNU2020XJ147* (BNU)
*L.occultata* Popov	China. Xnjiang, *Dan-Hui Liu BNU2020XJ088* (BNU)
*L.redowskii* (Hornem.) Greene	China. Gansu, *Dan-Hui Liu BNU2020GS038* (BNU)
*L.shanhsiensis* Kitag.	China. Shanxi, *Dan-Hui Liu BNU2020GS117* (BNU)
*L.tianschanica* Popov & Zakirov	China. Xinjiang, *Dan-Hui Liu BNU2019XJ346* (BNU)
*Rocheliabungei* Trautv.	China. Xinjiang, *Dan-Hui Liu BNU2021XJ076* (BNU)
*Pseudolappulasinaica* (A.DC.) Khoshsokhan, Sherafati & Kaz.Osaloo	China. Xinjiang, *Dan-Hui Liu BNU2021XJ095* (BNU)
